# Reflections on dynamic consent in biomedical research: the story so far

**DOI:** 10.1038/s41431-020-00771-z

**Published:** 2020-11-28

**Authors:** Harriet J. A. Teare, Megan Prictor, Jane Kaye

**Affiliations:** 1 Centre for Health, Law and Emerging Technologies, Faculty of Law, University of Oxford, Oxford, UK; 2 Health, Law and Emerging Technologies, Melbourne Law School, University of Melbourne, Carlton, VIC Australia

**Keywords:** Ethics, Medical ethics

## Abstract

Dynamic consent (DC) was originally developed in response to challenges to the informed consent process presented by participants agreeing to ‘future research’ in biobanking. In the past 12 years, it has been trialled in a number of different projects, and examined as a new approach for consent and to support patient engagement over time. There have been significant societal shifts during this time, namely in our reliance on digital tools and the use of social media, as well as a greater appreciation of the integral role of patients in biomedical research. This paper reflects on the development of DC to understand its importance in an age where digital health is becoming the norm and patients require greater oversight and control of how their data may be used in a range of settings. As well as looking back, it looks forwards to consider how DC could be further utilised to enhance the patient experience and address some of the inequalities caused by the digital divide in society.

## Introduction

Digital health on the whole has been slow to progress compared with the digitalisation of other sectors. In 2019, the Topol Review in the UK [1] outlined the opportunities for digital health within the UK National Health Service (NHS). Alongside the establishment of NHSX [2], the UK Government unit tasked with driving digital health strategy and policy, this marked a decisive moment to promote the use of digital tools to support healthcare, and by extension, biomedical research in the UK. Ensuring these digital tools have appropriate mechanisms for consent will be a crucial feature for their development. This paper provides a necessary reflection at a time when the use of patient interfaces is accelerating and diversifying in use. Approaches such as dynamic consent (DC) could prove integral to the further adoption of digital health strategies.

Since DC was first conceived, it has been prototyped in a number of projects and has become an example of how digital technologies can support and enhance existing procedures and relationships in healthcare and biomedical research. DC is an approach to informed consent that allows communication and engagement through a secure digital portal in ways that have not been possible before, with individuals being able to revisit and review consent decisions and preferences over time, as and when they choose. By using a digital platform, information can be presented in new ways, through video clips to reach broader and more diverse audiences, but also enabling participants to input their own information and complete online questionnaires. It is dynamic because it can be tailored to the research endeavour and the expectations of participants, as well as when consent and interactions are needed at different points along the clinical or research pathway.

This paper tracks the progress of DC from its introduction and early criticisms to its implementation and evaluation. We discuss the influences of external factors on the development of DC and the importance of digital consent approaches in the future, as data become even more central to address global health challenges.

## The changing context of biomedical research

Global investment in research infrastructure such as biobanks and data repositories has been instrumental in supporting open access policies. The requirement of informed consent has been significantly tested by these research agendas that have promoted a wide range of different secondary uses of samples and data [3]. Informed consent is regarded as central to voluntary participation in biomedical research to notify participants of the risks and benefits of taking part, and to explain what will happen during a study. Scholars have questioned whether participants can make a truly informed decision if they are not provided with specific details of the intended use of biological samples and data at the time of consent, which is impossible if the ‘future research’ is yet to be defined [4]. These concerns run in parallel to broader societal conversations around the appropriate use of data in the age of the Internet, in terms of access, identity, privacy, control, and individual rights. In response to the implications of these changes, the DC approach to consent was developed to allow participants to review and update their consent decisions over time and to be informed of the many research uses of their data and samples. DC aims to mitigate the risk that downstream research will be conducted without the donors’ knowledge by drawing on digital technologies to support an ongoing relationship between researchers and participants.

## The development of DC

The concept of DC was conceived, and the term coined, in 2008 in the Ensuring Consent and Revocation project, which aimed to enable individuals to turn consent decisions on and off ‘as easily as turning on a tap’ [5]. The motivation behind the project was to give individuals the digital tools to control the way that their data were being used so they could better protect their informational privacy. Biobanking, with its collection of samples and data for future use, was one of three project case studies, because of the consent challenges that were arising in biomedical research [6]. DC was designed to allow researchers to see in real-time what permissions were associated with the data they were accessing, and enabled participants to review and update their consent decisions over time using an online portal. Both of these capabilities could be tailored to the needs of the research project allowing flexibility depending upon the nature of the research.

Coincident to the development of DC has been a growth in electronic consenting tools, which allow PDF copies of consent forms to be shared via email or electronic signatures to be recorded. These approaches tend to provide an electronic version of a paper form, maintaining a static single point consent, and an electronic receipt, rather than allowing participants to change their mind over time, and thus do not support the fundamental behaviour change that DC espouses [7].

Electronic or ‘eConsent’ methods have been touted as having advantages over, or at least being comparable with, paper-based consent, yet uptake has remained slow. For instance, only 8% of biobanks surveyed in 2014 reported using eConsent although 75% were interested in doing so [8]. A recent scoping review identified that the absence of a leading commercial eConsent product has resulted in a ‘myriad of homegrown systems’, creating inefficiencies and inconsistent user interfaces [9]. There are other reasons that the adoption of eConsent solutions has been slow and that paper consent is still common. These include concerns about the authentication and validity of electronic signatures, data security, and ease of use relating to the ‘digital divide’ [9]. These concerns have had implications for the adoption of DC, as discussed further below.

## Criticisms of DC

DC’s development has occurred alongside the continuing reliance of biomedical research on paper-based systems for consenting, with engagement, and communication still generally restricted to face-to-face encounters. It is also not unusual for research participants to hear nothing about the results of a project due to the additional costs that further communication involves. In this context, the suggestion to implement DC has often been seen as a resource-intensive process, with concern that it could negatively impact research quality if participants frequently changed their mind, and thus dramatically altered the dataset over time.

Concerns were raised by Steinsbekk et al. that DC would be an unwanted burden for participants and researchers. ‘In a DC model, participants will be asked for consent continuously, simply because each new project is a new project. Thus, they will be asked to re-consent both for trivial and essential reasons, and often the formerʼ [10]. This criticism misunderstood the flexibility and adaptiveness inherent in digital tools and the DC approach. Steinsbekk et al. also argued that DC might reduce participation rates: ‘[b]eing confronted with the detailed complexity of biomedical research … it is likely that at least some people will struggle with feelings of falling short—that their own competence or knowledge do not suffice.’ As digital health has progressed, and technology has been introduced to support different aspects of biomedical research, these concerns have eased, with greater consideration of the opportunities that DC might provide.

## Participants’ views of DC

In 2013, focus groups were conducted to explore research participants’ views about DC, with participants recruited from three biobanks [11]. These provided important insight into the realities of some of these criticisms, particularly around the likelihood of participants changing their minds. Many of the focus group participants, all of whom had previously signed up to a biobank, had limited memory of the consent process. They were largely interested in the opportunity to review their consent decisions, but could not, necessarily, imagine wanting to change their minds. DC’s appeal lay in its provision of a two-way channel of communication with the research team, and the opportunity to receive updates about how the research was progressing. This led to a marked focus on communication and engagement as a key feature of DC.

## Implementation of DC

From 2008 onwards, a number of international projects began piloting DC and similar approaches. For example, the Italian-based CHRIS study (which commenced in 2011) [12–14], implemented ‘an interactive DC process for empowering participants’ autonomy’ and experienced notable resource benefits by adapting their recruitment approaches. The RUDY (UK rare diseases) study [15, 16], initiated in 2014, incorporated DC to build a partnership model with participants detailing their rare disease experiences. The USA patient organisation Genetic Alliance, based on its long experience of working with patient groups, launched Platform for Engaging Everyone Responsibly (PEER). This digital platform allowed registry participants to select their privacy and decision preferences using the Private Access software. Phase 1 began in 2015, with 15 teams selected to trial the platform [17]. In 2016, a US research group combined DC software with an education website to support a research biobank of residual dried bloodspot cards from new-born screening. Their pilot study evaluating a simulated consent portal found that a digital approach to consent should be explored further, given the positive response from participants [18].

PEER and RUDY both used a DC approach and function within a broader software solution to support their digital studies. Another approach would be to develop a standalone DC tool that could be integrated with other software. The Oxford-based SPRAINED study (2015–17) piloted such a DC approach with a small cohort of participants, linking DC software with a clinical trials management tool [19]. The DC software collected consent decisions and provided information and updates to participants. The consent decisions were then delivered to the clinical trials software to be reflected in the trial permissions. DC has also been considered within the context of the NHS, for use in electronic health records [20, 21]. InBank, at the University of Manchester, conducted qualitative research with patients, which demonstrated potential support for this approach, particularly relating to feedback of research results [22]. These early adopters started demonstrating the technological realities and feasibility of DC and the opportunities and challenges it might present to research teams and participants alike.

Over this period, scholarly discussion of DC has continued, with criticism and support ebbing and flowing [23]. Proponents of broad consent still consider DC to be too complicated, too resource heavy, and to lend itself to consent fatigue [24]. Other consent models alongside broad consent have been touted as possible alternatives, for example, meta-consent [25] or tiered consent [26]. Advocates have continued to argue that DC provides the opportunity to redress several challenges in biomedical research [27], whilst calling for more robust evaluation of different facets of the tool and its ability to meet changing legal requirements in different settings. It is possible that the same factors that have hindered the uptake of simple eConsent, outlined above, have also impeded the widespread adoption of DC notwithstanding its various potential benefits for researchers and participants.

## Changing research paradigms

Greater emphasis on large volumes of data has changed the way that research is conducted and raises specific issues that are relevant to consent. Anonymisation of data is often considered a tool to enable consent to be waived; indeed, anonymous data are considered exempt from the European General Data Protection Regulation (GDPR) [28] and other national regulatory schemes. However, it is becoming increasingly difficult to guarantee anonymity, particularly with the risk of anonymous datasets being made identifiable when combined with other data sources [29, 30]. Furthermore, the ability for data, supported by digital technologies, to flow easily from different settings and sectors means that it is technologically possible for data collected for one purpose to be used for another. Arguably, this places even greater emphasis on the need for water-tight governance practices that allow oversight and control for individuals [31–33].

DC has also been considered as an innovative approach to support group decision-making—an opportunity that will become increasingly important as genomic medicine, relevant to family members, becomes more common [34, 35]. This possibility has been explored in relation to indigenous communities, specifically considering the implications of biological samples gathered for genetic research [36]. During this time, research exploring the needs and interests of participants has continued. This includes work examining the need for informed withdrawal [37]; patient perspectives on sharing anonymised personal health data [22]; the return of results [38]; and the acceptability of interacting with health data online [39], which provided the foundation for the establishment of a Japanese RUDY study for myotonic dystrophy [40].

While these findings demonstrate the potential opportunity for DC to support patients and participants to interact with their data, there is still limited evidence surrounding how DC affects the patient experience. To this end, an evaluation approach has been proposed to allow projects using DC to better understand its impact on key outcomes, such as knowledge and understanding, decisional conflict, satisfaction with the research, and trust in the researchers [7]. This will be a crucial step in moving beyond the promissory discourse around DC, to measure empirically the benefits and drawbacks of the tool, allowing researchers themselves to make an informed decision whether to adopt it as a viable consenting approach.

## External factors that have influenced DC’s development

The development of DC was motivated by the growing need for individuals to have control of how their data (and samples) were being used, especially in the face of new technologies and research methods (although as outlined above, the opportunity for DC to support collective control in wider groups and communities is also being explored). This recognition of the need for control reflected a number of social developments, alongside the introduction of biobanking as a new research infrastructure. The rapid development of digital tools, following the establishment of the Internet and the unprecedented opportunity to connect and network, redefined expectations of privacy and security and emphasised the non-physical risks of research participation.

Digital tools provided individuals with access to information relevant to their health and care that challenge the traditional doctor–patient relationship. The revised role of patients within healthcare was central to the white paper entitled ‘Liberating the NHS: no decision about me without me’ [41] that followed the introduction of the Health and Social Care Act 2012 (UK). This followed growing pressure for patients and the public to be involved in decision-making surrounding research and clinical care—as cemented by the 1996 establishment of INVOLVE [42], the National Institute of Health Research body responsible for championing patient and public involvement (PPI).

Greater emphasis on PPI chimed with other developments, such as an increasing acknowledgement of patients as experts [43, 44], and a push for shared decision-making and partnership approaches in healthcare and biomedical research [4]. Consumer activism in healthcare internationally, which had been increasing in importance and visibility in the last few decades of the 20th century, provided the basis for these developments, as did a shift away from paternalism towards a greater respect for patient and participant autonomy, and the need for greater information [45, 46]. The evidence-based medicine movement similarly has prioritised consumer participation and engagement, not simply as recipients of information, but as co-producers [47, 48]. Coupled with the rise in the use of social media and a rapidly changing world where individuals are more closely connected to the companies and industries providing products and services, a more conversational society has emerged, with an expectation for responsiveness [1].

As has been mentioned, research involving biobank participants has shown that those involved in the focus groups appreciated the opportunity to change their mind, although do not necessarily think that they actually would [11]. This suggests a recognition of the importance of having control and oversight of their data. Questions still remain, however, about how prevalent these views are and the extent to which they are acted upon outside a focus group environment, in amongst other priorities that individuals might have. In the RUDY study [15, 16], the majority of participants have not updated their consent decisions as research has progressed, which could either mean that they are satisfied with their original decisions or that they have not been sufficiently motivated to access their consent choices to change them. This could suggest that oversight and control is more appealing conceptually than in practice. Research is currently underway to understand how research participants engage with DC in a real-world setting, and to explore this reality, to understand whether consent choices are high priorities. Arguably, if the research findings suggest that people are not motivated to engage, this may point to the need for creative mechanisms to encourage engagement, perhaps by incorporating decisions about consent into other necessary interactions with the health system instead of via a standalone portal.

New technologies in research and care were also influential during this time. Biobanking has already been cited as a prompt for the development of DC, including long-term longitudinal studies involving patients and healthy participants. Research projects involving much larger cohorts were also initiated, with the conviction that rapid progress could be made now that technology could support the collection of huge volumes of data [49]. This has been particularly significant in genomics, with population studies initiated throughout the world to sequence large numbers of participants, such as the 100,000 genomes project in the UK [50] and the Australian Genomics Health Alliance, which aims to lay the foundation for genomic medicine in Australia [51].

Aside from the scale of such research, genomics has several other important characteristics that have been highly influential in shaping research practices, including DC. Genomic research projects are often established within a clinical environment, with participants recruited as part of their clinical care. Increasingly, it is acknowledged that research findings within these projects may be of direct relevance to the healthcare of the participant and their family members. Thus, pertinent findings are fed back to the patient, often via their clinician, to help shape treatment decisions. This is in direct contrast to traditional studies that assure the patient there will be no direct benefit for them in taking part. This has necessitated detailed consideration of the implications of research findings for patients, including secondary or incidental findings, whether it is ethically appropriate to share them or withhold them, and how best to support patients in their decision-making [52, 53]. Digital tools, such as DC, may be influential in supporting these interactions, allowing patients to reflect on their decisions and review them over time.

## The future of DC

The use of health data will continue to be supported by digital technologies that enable collection, processing, and sharing on a large scale. If oversight and transparency continue to be valued, digital platforms that enable individuals to engage easily with decisions around how the data are being used, will continue to be needed.

The response to the GDPR has demonstrated how challenging this reality is. Privacy and cookie notifications on websites are improving the degree of transparency about the use of data, however the effectiveness of these pop-up notifications is questionable. Consent fatigue is one of the criticisms routinely levelled at DC, and there is a need for better evidence about whether, and how, people actually adopt and interact with such a tool.

A major advantage that DC has over websites requiring consideration of cookie settings and privacy policies is that it is centrally organised for a single project, with clear oversight of the different decisions that participants will be presented with over the duration of the project. There is then opportunity for different decisions to be prioritised, with technology allowing for these different priorities to be organised to support varied levels of engagement. For example, preferences around communications could be grouped together and allow for quick response, whereas a decision relating to participation in genome sequencing could be set up to mandate consideration before the participant would be able to progress to the next page. The main challenge with cookie and privacy notices is that they appear for all websites, and users risk becoming overwhelmed by the sheer volume and the repetition between websites. In future if it was possible for participants to have access to a single research participation site incorporating DC, which consolidated all their health data decisions across different projects and clinical care as well, this would further mitigate against consent fatigue and help to support meaningful engagement with the decision-making process.

When the GDPR was in draft stage, the research community in the UK petitioned for exemptions for research [54], given the emphasis placed on explicit consent for use. The right to be forgotten was also a cause for concern given the difficulty in tracing data once they have been shared and published. This discussion has provided further opportunity to understand the expectations that individuals have for data use, and the extent to which privacy and security will need to be supported within research contexts. It raises interesting questions surrounding the role of patients as consumers, particularly in light of the explosion of health apps and other self-monitoring tools that are relevant to patient care and which provide new sources of information available to patients to shape their understanding of health. These sources of ‘real-world evidence’ are being drawn into research and clinical care and will require tools to manage their use.

While paper-based tools have not been phased out entirely and will likely always play a role, digital health is rapidly advancing in all areas of healthcare delivery, from electronic check-in at GP surgeries and hospitals to the use of tablet computers in the clinic to capture patient data. Electronic patient records can now facilitate entirely electronic interactions across healthcare settings. These could further cement the role of patients, if they have real-time access to their record and are positioned as gatekeepers.

There is hope that digital technology will be an enabler for improving access. For example, the aforementioned Topol Review [1] highlighted the opportunity for digital health to lead to greater face-to-face interactions between doctors and patients. It would introduce efficiencies and free up doctors’ time for the activities that matter most. This assertion has been challenged, with the acceleration of digital health in response to COVID-19, as consultations with general practitioners have moved online, and digital tools have been rapidly introduced to support virtual patient care. However, it is too early to predict the lasting influence of these circumstances and which digital practices will remain in the long term.

DC provides the opportunity to improve the accessibility of information to a wider range of participants due to of the range of media and virtual interactions that it can support. However, this is only possible if individuals have access to the required technology and the motivation to engage with it. While broadband connectivity has improved over the past few years and more households have computers and/or smart devices [55], there is still a sizeable population that do not have access or cannot—or prefer not to—engage digitally. We have considered elsewhere the risk that the tool could ‘exacerbate exclusion and disenfranchisement’, further marginalising people who already face challenges in exercising their rights [56]. Ensuring there are mechanisms to support these groups, to mitigate the digital divide, is crucial.

## Conclusion

DC goes beyond an informed consent tool for biomedical research and could help individuals and groups navigate their entire online presence, with data from a variety of different areas being shared for a range of activities. For this to be a reality, several features need to be carefully considered, including when consent decisions are required, when individuals are prompted to review them, which data are held and stored, and where and how the consent decisions are connected with the data. This is more than just being a tool for decision-making, as it raises wider societal issues about how personal information should be controlled in the digital landscape and by whom.

Several questions remain to be addressed, including how to ameliorate some of the effects of the digital divide, and whether patients and participants, in the reality of their everyday lives, want to take responsibility for oversight and control that a DC interaction allows, given the demands it might place upon the individual to engage.

In 2020, the COVID-19 pandemic has demonstrated the significance and pervasive impact that health-related data will have on our future. Finding ways to enable people to exercise control over these data in ways that is acceptable for them is important. Perhaps, it is time for the development of health dashboards that can help us to manage all aspects of our healthcare, including decisions about data relating to clinical care, involvement in research, social media, and real-world data. The development of DC for biomedical research, in response to broader societal concerns, demonstrates that health data cannot be treated in isolation. While the development of digital health tools may have trailed behind the digitalisation of other sectors, it is impossible for them not to be influenced by the experiences and expectations created more broadly. A digital consent tool is therefore the obvious direction of travel. The groundwork laid down through successive projects will help ensure that this tool is fit for purpose.
